# Selective IgA Deficiency and Allergy: A Fresh Look to an Old Story

**DOI:** 10.3390/medicina58010129

**Published:** 2022-01-15

**Authors:** Bianca Laura Cinicola, Federica Pulvirenti, Martina Capponi, Marta Bonetti, Giulia Brindisi, Alessandra Gori, Giovanna De Castro, Caterina Anania, Marzia Duse, Anna Maria Zicari

**Affiliations:** 1 Department of Maternal Infantile and Urological Sciences, Sapienza University of Rome, 00185 Rome, Italy; martina.capponi88@gmail.com (M.C.); martabonettimb@gmail.com (M.B.); giulia.brindisi@uniroma1.it (G.B.); alessandra.gori85@gmail.com (A.G.); giovanna.decastro@uniroma1.it (G.D.C.); caterina.anania@uniroma1.it (C.A.); marzia.duse@fondazione.uniroma1.it (M.D.); annamaria.zicari@uniroma1.it (A.M.Z.); 2 Department of Molecular Medicine, Sapienza University of Rome, 00185 Rome, Italy; 3 Primary Immune Deficiencies Unit, Department of Internal Medicine and Infectious Diseases, Azienda Ospedaliera Universitaria Policlinico Umberto I, 00185 Rome, Italy; federica.pulvirenti.md@gmail.com; 4 Department of Translational and Precision Medicine, Sapienza University of Rome, 00185 Rome, Italy

**Keywords:** SIgAD, allergy, immunology, infections, asthma, microbiota

## Abstract

Selective IgA deficiency (SIgAD) is the most common human primary immune deficiency (PID). It is classified as a humoral PID characterized by isolated deficiency of IgA (less than 7 mg/dL but normal serum IgG and IgM) in subjects greater than 4 years of age. Intrinsic defects in the maturation of B cells and a perturbation of Th cells and/or cytokine signals have been hypothesized to contribute to SIgAD pathogenesis. The genetic basis of IgA deficiency remains to be clarified. Patients with SIgAD can be either asymptomatic or symptomatic with clinical manifestations including allergy, autoimmunity and recurrent infections mainly of the respiratory and gastrointestinal tract. Studies analyzing allergy on SIgAD patients showed prevalence up to 84%, supporting in most cases the relationship between sIgAD and allergic disease. However, the prevalence of allergic disorders may be influenced by various factors. Thus, the question of whether allergy is more common in SIgAD patients compared to healthy subjects remains to be defined. Different hypotheses support an increased susceptibility to allergy in subjects with SIgAD. Recurrent infections due to loss of secretory IgA might have a role in the pathogenesis of allergy, and vice versa. Perturbation of microbiota also plays a role. The aim of this review is to examine the association between SIgAD and atopic disease and to update readers on advances over time at this important interface between allergy and SIgAD.

## 1. Introduction

Selective IgA deficiency (SIgAD) is the most common primary immunodeficiency disease with an estimated incidence varying in Caucasians from 1:3000 to 1:150, depending on the study population [1,2]. It is defined when IgA serum level is equal to or below 7 mg/dL with normal IgG and IgM levels, in patients older than 4 years of age with an otherwise normal immune system [3]. When serum IgA level is more than 7 mg/dL, but <2 standard deviations (SD) for age, it is defined as partial or probable IgA deficiency [2]. Although SIgAD is rather common, the underlying pathogenetic mechanism remains largely unexplored [4,5]. Besides the deficiency of serum IgA, patients with SigAD suffer also from a decrease of secretory IgA. This leads to colonization and penetration by pathogenic bacteria, making these patients prone to recurrent infections. The loss of mucosal IgA might also facilitate the passage of aeroallergens and food antigens, promoting the development of allergies [6].

It has been estimated that allergies can be the first manifestation of SigAD in up to 40% of cases [7]. Therefore, the suspicion of SIgAD should heighten not only in patients with recurrent infections but also in those with atopic manifestations. Here, through this narrative review, we aim to examine and update readers on this important association between SIgAD and atopic disease. At present, based on our research, 21 studies analyzing allergy in cohorts of SIgAD patients present data on overall 534 patients. Asthma was the most frequently described allergic manifestation in SIgAD followed by rhinitis and atopic dermatitis, with allergy being the first symptom of SIgAD in many cases. Hypotheses on the pathogenesis of allergy in SIgAD include high penetration of allergens through the mucosa due to defective serum and mucosal IgA, facilitating the contact of allergens with the local respiratory and gastrointestinal mucosal immune system. Moreover, the increased susceptibility to infection and the dysregulation in the homeostasis of the intestinal microbiome may also contribute to allergy pathogenesis.

## 2. IgA Structure and Function

IgA is a class of immunoglobulins characterized by the presence of an alpha heavy chain. In humans, there are two subclasses of IgA—IgA1 and IgA2—that differ in the structure of the hinge region and in the number of the glycosylation sites. IgA1 is the predominant subclass in the serum, accounting for as much as 90%, while in mucosal tissue both subclasses are equally distributed [8]. In humans, IgA present as monomeric, prevalent in the serum, and polymeric, found in secretions such as breast milk, sputum and bronchial and gastrointestinal fluids. Polymers of secretory IgA, mainly dimers, are covalently linked by a J-linking chain and secreted into the mucosal surface with their secretory complement, which protects them from the proteolysis of bacteria in the intestinal lumen [9]. As for all the Ig classes, IgA are produced by B lymphocytes that have differentiated into plasma cells. Secretory IgA is produced locally at the level of gut-associated lymphoid tissue (GALT), mainly as IgA2 by both T-dependent and T-independent mechanisms [10,11], while serum IgA is produced by the bone marrow, mainly in the monomeric form IgA1 [9].

**Figure 1:**
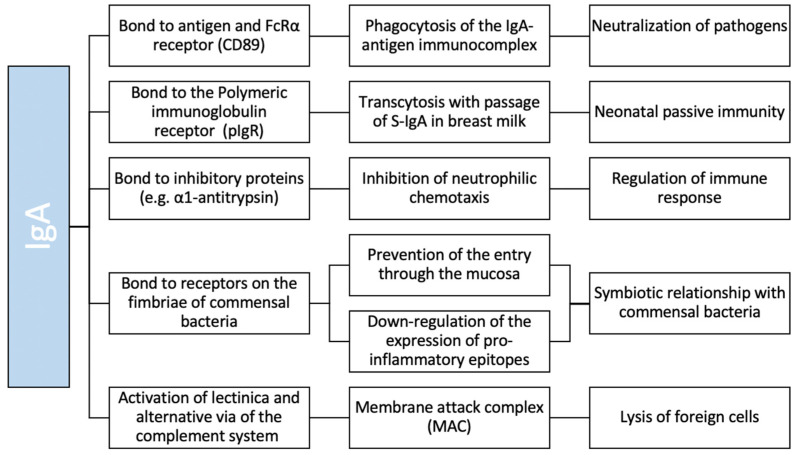
Schematic function of IgA.

In mucosal areas such as the gastrointestinal, genitourinary and respiratory tracts, IgA is the most abundant antibody class [9], playing a key role in keeping a tight balance by tolerating commensals and harmless (as food) antigens on the one hand and providing protection against harmful pathogens on the other hand. Mucosal IgA protects the host by diversifying the microbiota and neutralizing toxins and viruses without causing inflammation, by the inability of activating the complement cascade. Moreover, it blocks colonization and penetration of pathogenic bacteria by binding with receptors on the fimbriae, clearing unwanted particles and promoting sampling of antigens [12]. In serum, IgA is the second most predominant antibody class after IgG. In contrast to mucosal IgA, the roles of serum IgA are relatively unknown [13]. By binding the FcRα receptor on the phagocyte’s membrane, serum IgA allows the phagocytosis of the IgA-antigen immune complex. Moreover, IgA suppresses neutrophil chemotaxis through binding to inhibitory proteins such as α1-antitrypsin [14] (Figure 1).

## 3. Selective IgA Deficiency (SIgAD)

### 3.1. Definition and Incidence

As stated by the European Society for Immunodeficiencies (ESID), the criteria for the definitive diagnosis of SIgAD include patients greater than 4 years of age who have serum IgA levels of less than 7 mg/dL (0.07 g/L) but normal serum IgG and IgM (also defined as isolated IgA deficiency), where other causes of hypogammaglobulinemia have been excluded, and there is normal IgG antibody response to vaccination. The diagnosis is “probable” when the serum IgA levels are at least 2 SD below normal for age with the same other criteria as for the definitive diagnosis [15]. Causes of isolated IgA deficiency other than SIgAD include drugs, infections, monogenic diseases and chromosomal abnormalities (Table 1) [16].

The incidence of SIgAD presents considerable variability when comparing different ethnic groups. In the Caucasian population SIgAD is the most common primitive humoral immunodeficiency with an incidence varying from 1:150 to 1:3000, while in Asian populations its incidence is significantly lower (1:4000 in China and up to 1:18,000 in Japan) [1,2]. However, since many patients affected by SIgAD are asymptomatic, and due to the lack of screening programs for immunodeficiency, the real incidence of SIgAD can be highly underestimated [17].

### 3.2. SIgAD Pathogenesis

SIgAD is a heterogeneous disorder with the underlying pathogenetic mechanism remaining largely unexplored [9]. The considerable variability in clinical manifestations suggests multiple mechanisms contributing to the pathogenesis of SIgAD [6].

Intrinsic defects in the class switch recombination (CSR) mechanism and in the maturation of B cells have been reported, with decreased levels of peripheral class-switched memory B cells who cannot differentiate into IgA-secreting plasma cells [18]. 

IgA CSR occurs by engagement of different receptors, transcription factors and cytokines such as the toll-like receptor, B cell receptor (BCR), nitric oxide (NO), retinoic acid (RA), IL-6, TACI, BAFF, A proliferation-inducing ligand (APRIL) and thymic stromal lymphopoietin [11,19,20,21,22,23,24]. Therefore, disturbed signaling pathways or receptor defects of each of these components might be involved in patients living with SIgAD.

In addition to intrinsic maturation defects of B lymphocytes, dysfunction of T helper (th) and regulatory T lymphocytes (T regs) has also been observed in SIgAD. Indeed, defective antibody production may be related to a decreased or compromised T helper activity as evidenced by a recent study where adult and pediatric patients had reduced Th1 and Th17 cells compared to controls [25]. Moreover, T regs expressing the transcription factor Foxp3 play a critical role in the control of immune homeostasis, including the regulation of humoral immunity. Among them, T-follicular regulatory cells (Tfr), a specialized subset of T regs, help in the control of T-follicular helper (Tfh) cell-driven germinal center (GC) responses [26]. A decreased number of T regs could contribute to pathogenesis of SIgAD [27].

### 3.3. Inheritance and Genetics

SIgAD generally occurs sporadically, but autosomal recessive, autosomal dominant and sporadic transmission patterns have all been observed [28]. It is estimated that the possibility of inheriting the disease in the family is about 20% [29].

Among identified genetic defects, mutations in transmembrane activator and calcium-modulator and cyclophilin ligand interactor (TACI, TNFRSF13B) have been reported in 7–10% of Common Variable Immunodeficiency (CVID) patients and 13% of SIgAD carried at least one mutated TNFRSF13B allele [30]. TACI, a B-cell surface ligand for BAFF and APRIL, has a role in B-cell function, development and tolerance. The same TACI mutation may be present in individuals with either IgA deficiency or CVID in the same family [24]. However, it is controversial whether TACI mutations have a cause–effect relationship with IgA deficiency or CVID [2,30]. Because of the familial occurrence of SIgAD and CVID and the possible progression from SIgAD to CVID, a common genetic basis for the two disorders has been proposed [31].

With regard to MHC associations, a predisposing locus was found on chromosome 6 in the MHC region of classes II and III [32] and the DR/DQ locus was reported as the most involved in the genetic predisposition to the disease [33]. A more detailed genetic analysis confirmed the association between SIgAD and HLA A1-B8-DR3-DQ2 (extended haplotype 8.1) hypothesizing that this may confer a higher risk of developing the disease [34].

The set of these findings could be at the basis of both ethnic differences and the association with autoimmune diseases (frequently found in patients with SIgAD). Indeed, some studies have shown that subjects with autoimmune diseases have a higher frequency of HLA B8 [35]. In line with this, SIgAD patients with autoimmune disorders (B lymphocytes or T reg lymphocytes) and severe infections (also linked to IgG subclass deficiency or specific antibody deficiency) are at a higher risk of developing CVID. In 2010 an association was found between SIgAD and an amino acid variant of the IFIH1 gene, associated with diabetes mellitus 1 (DM1) and systemic lupus erythematosus (SLE), supporting the hypothesis that autoimmune mechanisms may contribute to the pathogenesis of SIgAD [36].

### 3.4. Clinical Manifestations

Patients with SIgAD clinically range from asymptomatic patients, who are diagnosed coincidentally during a laboratory screening, to symptomatic patients with heterogeneous clinical phenotypes and severity of the disease: recurrent infections, mainly of the respiratory and gastrointestinal tract, are the most common finding [28]. Allergy is a quite common clinical complication affecting more than 30% of patients. Finally, autoimmune diseases are associated with SIgAD in up to 20% of subjects [5]. This heterogeneity in clinical symptoms may be related to the different combination of etiological agents. The lack of serious infection in some patients may in some cases be due to a compensatory increase in secretory IgM [37]. The clinical course of the disease is generally favorable in most patients, being influenced by the clinical manifestation. However, progression to CVID is observed in about 5% of patients [31].

The most frequent and relevant clinical manifestations are recurrent infections, especially those affecting the respiratory system and the gastrointestinal tract [16,28,38]. Infections in adults occur more frequently as rhinosinusitis or lung infections while otitis media is less common [39,40], whereas in children the main infections are pharyngotonsillitis, otitis, bronchitis, sinusitis and, less frequently, pneumonia [41,42,43]. 

As a consequence of recurrent or chronic infections, some patients may present with organ damage such as bronchiectasis [43,44,45], especially in those with concomitant IgG2 [46] or IgG4 subclass deficiency and/or limited antibody response to the pneumococcal polysaccharide [47].

Impaired antibody response to vaccinations are considered exclusion criteria for SIgAD [15]. Few studies have explored the issue of post-immunization response in real life, concluding that there was no relationship between the history of recurrent infections and the absence of protective antibody titers against vaccination, including the ones for pneumococci [41,48].

However, evaluation of antibody response can be indicated for patients with SIgAD and a history of recurrent respiratory infections, IgG2 subclass deficiency, or low specific antibody levels, considering also that ESID revised criteria reclassified lack of response to polysaccharide vaccines as a separate humoral immunodeficiency [49].

Recurrent infections of the intestinal tract are also common due to alteration of the protective IgA-mucosal barrier of the gastro-intestinal tract, which facilitates the adhesion of pathogens to the epithelium leading to proliferation and establishment of the parasitosis, mainly Giardia Lamblia [50]. Infection by Helicobacter Pylori, Salmonella and Campylobacter are also common [39].

IgA deficient patients are also more susceptible to non-infective gastrointestinal diseases such as lactose intolerance, celiac disease, inflammatory bowel disease, nodular lymphoid hyperplasia and tumors [2]. Malabsorption can also occur, usually secondary to the structural damage of the intestinal villi or the onset of celiac disease [51]. Due to changes in the intestinal wall (damage to the mucosa, atrophy of the villi, alterations in mucosal clearance), food molecules introduced with the diet can pass through the sub-epithelial tissues and the submucosa causing the formation of antibodies and leading to the development of food intolerances or allergies [52].

A wide variety of autoimmune diseases are also associated with SIgAD, including thrombocytopenic purpura, autoimmune hemolytic anemia, type 1 diabetes mellitus, rheumatoid arthritis, systemic lupus erythematosus, Graves’ disease, celiac disease and vitiligo [53,54].

It has been hypothesized that the absence of IgA in the serum may allow the entry of cross-reactive antigens into the circulation leading to the development of autoimmune reactions [6].

In some patients the presence of anti-IgA antibodies that can cause anaphylactic reactions in those receiving blood product transfusions has also been found. For these patients, the use of blood products must be carefully evaluated [55].

Patients with IgA deficiency have been reported to be at higher risk for malignancies, especially gastric and colon adenocarcinoma [56] and lymphoproliferative diseases [57]. However, recent studies do not confirm the data between SIgAD and malignancy, making the association still not conclusive [58].

## 4. SIgAD and Allergy

Allergy is a hypersensitivity reaction of the immune system to a specific allergen and can be considered a deregulated form of immunity [59]. It is a chronic disease affecting over 20% of people worldwide but its prevalence is influenced by many factors such as country, ethnic background and season [60].

It is widely accepted that SIgAD is associated with allergy and various atopic manifestations including allergic rhino-conjunctivitis, asthma, urticaria, food allergy and atopic dermatitis (AD) [6,38]. However, there is still controversy on the true prevalence of allergy and its manifestations in SIgAD, arguing whether it can be considered a comorbidity or a consequence of the disease. Indeed, our analysis of published data on association between allergy and SIgAD is not conclusive, due to the heterogeneity of age, ethnicity and sample size of the study population and the allergic manifestations considered.

Moreover, the hypothesis of a higher risk for allergy in SIgAD patients in comparison to the general population is difficult to demonstrate due to the high prevalence of both these clinical conditions. Thus, allergy in SIgAD patients in comparison to the general population remains to be proven [61,62].

Studies analyzing allergy on SIgAD patients showed prevalence ranging from 13 to 84% (Table 2), supporting in most of the cases the relationship between SIgAD and allergic diseases. 

In an earlier study, Buckley et al. reported the frequency of atopy in children and adults with SIgAD accounting for 58% [63]. These data were further confirmed by Klemola et al., who reported symptoms of atopic diseases in 50% of children with SIgAD [64].

In more recent studies, allergic manifestations were recorded in 43.2% of 118 Turkish children with SIgAD [65]. In Italy, two studies on independent pediatric cohorts reported that 39% of 184 and 38% of 103 patients living with SIgAD were also diagnosed with allergy [42,66].

It is worth mentioning that in many cases allergy is the first symptom of SIgAD, and, in some cases, it is the only symptom of disease [67]. Indeed, it is also reported that up to 25% of SIgAD patients are diagnosed during an allergology clinical assessment [6]. Moreover, patients can develop new allergic manifestations during follow-up suggesting that periodic allergic assessment in SIgAD patients is warranted, especially when a positive family history for allergies is present [42,68].

The association between SIgAD and allergy was found for various allergic manifestations, the most commonly described of which are asthma, allergic rhinitis, allergic conjunctivitis, urticaria, atopic dermatitis and food allergy, even if the exact prevalence of each symptom and of one clinical manifestation or another varies among the studies reported. 

Among the most recent studies with a larger sample, on 39 over 103 pediatric patients Moschese et al. found allergic rhinitis in 18.45%, atopic dermatitis in 12.6% and allergic asthma in 10,67% [66]. 

In the study of Erkoçoğlu et al. involving 81 pediatric patients, 45.7% had an allergic disorder of whom 34.6% had asthma, 27.2% had allergic rhinitis and 11.1% had eczema [69]. Similarly, in the study of Živković et al. there was a higher prevalence of allergic diseases, specifically allergic rhinitis (59%), asthma (57,9%) and atopic dermatitis (15,8%) in children with low IgA compared to controls [70]. 

With respect to the type of sensitization to skin prick test (SPT), only two studies were found reporting the type of allergen involved in the allergic manifestations of patients with SIgAD [42,67]. Moreover, in one of them the specific type of symptoms that relate to the SPT positivity are not even defined. It is known that the prevalence of a specific allergen varies among countries [71], however, in both studies, Dermatophagoides was the main allergen identified. 

### 4.1. Atopic Dermatitis (AD)

AD is the most common skin disorder in childhood, with a reported prevalence of up to 20% [72]. As for the general population, there is a huge variety in the prevalence of AD in SIgAD, possibly due to heterogeneity on diagnostic criteria for AD among studies and ethnic diversity [61,73].

A study on 102 SIgAD by Gualdi et al. recorded a prevalence of AD in SIgAD accounting for 57.84%, although only 10.17% of those patients had elevated IgE [74]. A lower prevalence (4.6%) of AD in SIgAD was reported by Magen et al., even if significantly higher than that recorded in the control group [61].

### 4.2. Food Allergy

To the best of our knowledge, few studies analyzed the overall prevalence of food allergies as a specific clinical manifestation of atopy in patients with SIgAD. 

Different studies reported that IgA tended to be at the low-normal range or below in food allergic patients [75,76]. Data on food allergy and SIgAD are not conclusive. The prevalence of food allergy in patients with PIDs was examined using the US Immunodeficiency Network (USIDNET), recording a lower prevalence of food allergy in SIgAD in comparison to the general population [77,78]. The data were further confirmed by other studies focused on SIgAD cohorts [39,43,65,69,79]. On the other hand, Aghamohammadi et al. recorded a prevalence of food allergy in SIgAD of 22%, reporting egg, cow’s milk and hazelnut as causative allergens [67]. Another study reported an increased risk of food hypersensitivity over time among children with SIgAD [62].

### 4.3. Asthma and Recurrent Infections

It has been reported that asthma and allergic symptoms are significantly more common in adults [80] and children [81] with low IgA levels, although within the normal range. The same group of children, aged 18 to 23 months, also displayed a greater risk of infection [81]. Moreover, a high number of IgA-specific salivary antibodies has been connected to a lower risk of late-onset wheezing in sensitized infants [82]. Indeed, insufficient protection provided by IgA deficiency on the respiratory mucosa might predispose one to develop bronchial hyperresponsiveness and asthma [83].

However, conflicting results arise from data on the relationship between asthma and SIgAD. The prevalence of asthma among SIgAD patients was high in the majority of studies [67,69,70,83,84]. However, no difference in prevalence was found comparing SIgAD patients and the control group in the case-control by Jorgensen et al. [39]. 

Asthmatic patients are more likely to be diagnosed as having of SIgAD/CVID than non-asthmatic subjects [85]. Moreover, asthma has shown to be less responsive to standard treatment in PID, leading to the development of a chronic respiratory disease [86]. The results of Živković’s study show how children with SIgAD have significantly reduced lung function, which was related to the severity of SIgAD [70]. 

Recurrent infections of the respiratory tract are the most common clinical manifestation in primary immunodeficiencies, including SIgAD [28,42,66,87]. In this clinical setting, the presence of asthma is a worsening factor with respect to the number of respiratory infections [85] as the chronic inflammation of the airways facilitates the adherence of pathogens to the respiratory epithelium [88,89]. The data was confirmed by De Moraes et al. who showed the association between the degree of asthma control and recurrent airway infections as well as deficiencies within the immune system in a group of 41 severely asthmatic children [90]. 

**Table 2:** Prevalence of allergy and allergic specific manifestations in published SIgAD study cohorts.

Authors, Year (References)	Country	N of pts	Type of pts (P/A)	Allergy, *n* (%)	Controls Included	Asthma, *n* (%)	Rhinitis, *n* (%)	Atopic Dermatitis, *n* (%)	Urticaria, *n* (%)	Food Allergy, *n* (%)	Sensitisation, *n* (%)
Janzi, 2009 [62]	Sweden	14	P	4 (29)	Yes	1 (7)	NA	3 (21)	NA	4 (29)	4 (29)
Aghamohammadi, 2009 [7]	Iran	37	P + A	31 (84)	No	19 (51)	16 (43)	18 (49)	NA	8 (22)	31 (48)
Shkalim, 2010 [91]	Israel	63	P	20 (32)	No	15 (24)	8 (13)	2 (3.2)	2 (3)	NA	NA
Erkocoğlu, 2017 [69]	Turkey	81	P	37 (46)	No	28 (35)	22 (27)	9 (11.1)	NA	1 (1)	18 (22)
Aytekin, 2012 [65]	Turkey	118	P	51 (43)	No	25 (21)	27 (23)	16 (14)	6 (5)	2 (2)	NA
Lougaris, 2019 [42]	Italy	184	P	72 (39)	No	NA	NA	NA	NA	NA	132 (72)
Moschese, 2019 [66]	Italy	103	P	39 (38)	No	11 (11)	19 (18)	13 (13)	NA	NA	NA
Dominguez, 2012 [43]	Spain	330	P	62 (19)	No	21 (6)	10 (3)	12 (4)	NA	14 (4)	NA
Edwards, 2004 [41]	US	127	P + A	16 (13)	No	NA	NA	NA	NA	NA	NA
Plebani, 1987 [92]	Italy	80	P	20 (25)	No	16 (20)	NA	5 (6)	NA	NA	NA
Živković, 2019 [70]	Croatia	95	P	NA	Yes	55 (58)	56 (59)	15 (16)	NA	NA	NA
Jorgensen, 2013 [39]	Iceland	32	A	15 (47)	Yes	6 (9)	12 (38)	21 (66)	2 (6)	2 (6)	NA
Abolhassani, 2015 [84]	Iran	57	P	32 (56)	No	17 (30)	NA	NA	NA	NA	NA
Gualdi, 2015 [74]	Italy	102	P	NA	No	NA	NA	59 (58%)	NA	NA	NA
Magen, 2017 [61]	Israel	374	P + A	NA	Yes	NA	NA	16 (4%)	NA	NA	NA
Papadopoulou, 2005 [83]	Greece	20	P	11 (55)	No	17 (85)	NA	NA	NA	NA	11 (55)
Wang, 2020 [93]	China	43	P + A	6 (14)	No	0	0	0	0	0	NA
Delavari, 2020 [79]	Iran	116	P	33 (28)	No	11 (9)	5 (4)	6 (5)	2 (2)	6 (5)	NA
Jacob, 2008 [54]	Brazil	126	P + A	61 (48)	No	NA	NA	NA	NA	0	NA
Burgio, 1980 [68]	Italy	50	P	12 (24)	No	NA	NA	NA	NA	NA	NA
De Laat, 1991 [94]	Netherlands	40	P	12 (30)	No	8 (20)	6 (15)	5 (13%)	NA	NA	NA

Abbreviation: P, pediatrics; A, adults; NA, not available.

While Erkocoğlu et al. showed no significant difference in the prevalence of allergic disorders between the SIgAD patients with and without recurrent infections [89], another study reported a higher prevalence of respiratory tract infections among patients with SIgAD and allergy compared to SIgAD without any allergic manifestation [79]. An association to bronchial hyperresponsivity was detected among children sensitized to dust mites [83].

In light of the association between allergy/asthma and recurrent infections in SIgAD, early detection and management of respiratory disorders is essential to prevent severe complications.

### 4.4. Etiopathogenesis of Allergy

It has been postulated that serum IgA helps prevent the circulation of allergens and that secretory IgA plays a protective role on the mucosal surface against allergic disease [95]. Indeed, their opsonizing activity reduces the anchoring, penetration and invasion of antigens through the mucosa, preventing the absorption of food and aeroallergens and their contact with the local respiratory and gastrointestinal mucosal immune system [9,95,96]. As a consequence, the absence of IgA could reduce the protective effect on mucosal surfaces of antigen exclusion of microbial components due to increased permeability at that level and impair the mucosal immunity barrier, leading to the increased susceptibility to infection and sensitization against such antigens observed in these patients [80,81,97,98,99].

A demonstration of this mechanism comes from the evidence of the maternal secretory IgA role that, after transmission to the newborn through breast milk, ensures the complexation and subsequent removal of bacteria in the infant’s gut. The lack of the IgA antibodies in mothers with SIgAD increases the exposure to food allergens and consequently also the likelihood of developing food allergy in the newborn, as shown in a study focusing mainly on cow’s milk allergy [100].

Predisposition to allergy could also be a result of the inability to induce the inhibitory signaling by activating Fc receptors, due to decreased level of monomeric serum IgA, which, consequently, causes overactivation of the immune system [54,101]. Another hypothesized mechanism is the deficiency of TGF-beta response which can induce IgA synthesis and inhibit proliferation of Th2 cells [102].

The majority of patients with deficiency of secretory IgA have substitution with secretory IgM. However, in SIgAD allergic patients, proper mucosal compensation of IgM might not be assured, allowing antigens to pass through the mucosa and predispose one to developing allergy in the gut and respiratory tract [103].

Concerning eczema and food allergy, it was also found that serum IgA plays a role in suppressing IgE-mediated food allergy. In the study conducted by Strait et al. concerning IgE-mediated systemic anaphylaxis induced by ingested allergens, it has been found that both serum antigen-specific IgG and IgA antibodies can protect against severe IgE-mediated allergic reaction [104]. Thus, decreased serum IgA antibody levels might predispose one to increased intestinal mucosal permeability and absorption of ingested antigens, thus increasing the risk of severe food allergy [105].

Recent years have shown that there is a strong connection between the microbiota and allergy development. Since dysfunctions in IgA biology are associated with the diseases described above such as recurrent infections and allergies, it is reasonable to assume that a dysfunction of the IgA-microbiota axis may contribute to the development of these diseases/comorbidities [106].

Recently, the importance of IgA in regulating the homeostasis of the intestinal microbiome has been highlighted, but the scientific literature specifically evaluating this interaction is lacking. 

There are conflicting studies on whether SIgAD is associated with substantial/significant changes in gut microbial ecology. In a study by Fadlallah et al., metagenomic analysis revealed the presence of minimal alteration in the gut microbiota characterized by the predictable expansion of some pathogenic species and, unexpectedly, a less pronounced than expected depletion of some beneficial symbionts [107].

A plausible explanation given to the phenomenon is that the partial compensatory IgM response may preserve the diversity of the microbiota [108]. Contrary to this hypothesis, a subsequent study by Catanzaro et al. showed that patients with SIgAD exhibit significant dysbiosis of the gut microbiota even in the presence of an effective compensatory IgM response [109]. This study showed that secretory IgA in healthy controls targets a specific subset of germs in the gut microbiota whereas compensatory IgM has a lower specificity than IgA and targets a broader subset of microbial species. Therefore, IgA plays a critical and non-redundant role in controlling the composition of the gut microbiota in humans, and its evolution is linked to the maintenance of a diverse and stable gut microbial community. A recent study showed that patients with SIgAD exhibit a compensatory IgG response in the systemic circulation, convergent and synergistic with the secretory IgA response [110]. Finally, another metagenomic study showed that patients with SIgAD have a gut microbial profile characterized by a lower richness in terms of OTUs (Operational Taxonomic Units) and diversification compared to healthy controls and an increase in opportunistic bacteria such as Escherichia coli [111]. 

The data and studies available regarding the analysis of the microbiome of the nasal and oral cavities are even more limited. A study by Maria José de la Cruz Peña [112] showed that in a small cohort of non-pediatric patients with SIgAD, the change in the composition of the microbiome at the level of the oral mucosa was minimal or moderate even though a significant depletion of bacterial cells was present. However, it is conceivable to think that SIgAD can influence the respiratory tract microbiota, since a disrupted IgA barrier may lead to invasion and replication of aspirated foreign antigens. Since studies linking SIgAD and the microbiota are scarce and only a few years old, further research is needed to determine the effects of microbiota on the pathogenesis of the disease and on the complications connected to SIgAD, especially infections and allergy. 

## 5. Conclusions

The review investigated the role of IgA in the development of atopic disorders and the prevalence of allergy among studies on SIgAD. Despite debate still being open and results not being uniform, the overall revelation is that patients with IgA deficiency are at an increased risk of atopic disease. Prognosis is generally good and treatment for atopic manifestations is based on current standards of care for specific diseases [16,28]. However, concurrent presence of allergy, recurrent infections and autoimmunity and the possible development of a more severe allergic phenotype and complications can lead to a variable prognosis affecting quality of life of these patients. It is recommended that clinicians further investigate the clinical history of allergy to establish appropriate prophylaxis measures and/or targeted therapies that would be more effective, especially if started promptly, bearing in mind that today there is no treatment for SIgAD but only for associated diseases. 

## Figures and Tables

**Table 1:** Causes of isolated IgA deficiency other than SIgAD.

Drugs	Antimalarial Agents
	Carbamazepine
	Valproate
	Glucocorticoids
	Fenclofenac
	Gold salts
	Penicillamine
	Sulfasalazine
Infections	Congenital Rubella
	Congenital Cytomegalovirus Infection
	Congenital Toxoplasma Gondii Infection
	Epstein-Barr Virus
Monogenic disease	Ataxia-telangiectasia
	Wiskott-Aldrich Syndrome
	X-linked lymphoproliferative disease
	Transcobalamin II deficiency
Chromosomal abnormalities	Monosomy 22
	Deletion syndrome of chromosome 18q
	Trisomy 22
	Trisomy 8
